# Managing restricted patients in acute, non-secure in-patient services: clinical, ethical and resource implications of long waits for a response from the Ministry of Justice

**DOI:** 10.1192/bjb.2021.53

**Published:** 2022-08

**Authors:** Gunjan Sharma, Penelope Brown, Ijaz Ur Rehman, Edward Chesney

**Affiliations:** 1 South London and Maudsley NHS Foundation Trust, UK; 2 King's College London, UK

**Keywords:** Human rights, forensic mental health services, psychiatry and law, ethics, in-patient treatment

## Abstract

**Aims and method:**

In-patients subject to Section 37/41 of the Mental Health Act 1983 (MHA) require permission from the Ministry of Justice (MoJ) for leave, transfer and discharge. This study aimed to quantify the time spent waiting for the MoJ to respond to requests, using data on restricted patients recalled to a non-forensic unit over 8 years.

**Results:**

Eleven admissions were identified. The mean total time waiting for response was 95 days per admission, with an estimated cost of £40 922 per admission.

**Clinical implications:**

Current procedures may contribute to considerable increases in length of stay. This goes against the principles of the MHA, as non-secure services rarely provide the range of interventions which justify prolonged admission. We suggest several ways to resolve this issue, including broadening the guidance for the use of voluntary admissions and civil sections, and allowing clinicians to make decisions on leave and transfer where there is little risk.

## Restricted Patients and the Mental Health Act

Mentally disordered offenders at the Crown Court can be diverted to hospital for treatment at the point of disposal via a hospital order (Section 37 (s37) of the Mental Health Act 1983 (MHA)). In cases where the judge deems it necessary to protect the public from serious harm, a restriction order under s41 of the MHA can also be imposed by the court.^1–3^ Individuals detained under s37/41 of the MHA are referred to as ‘restricted patients’. When detained in hospital for treatment, restricted patients receive routine clinical care under the authority of their responsible clinician. Unlike the case of non-restricted patients, however, the responsible clinician does not have the power to decide on certain matters, including granting leave of absence under s17 of the MHA (s17 leave), transferring the patient to another hospital or discharging the patient. These powers are held by the Secretary of State for Justice and are exercised by officers in the Mental Health Casework Section (MHCS) within the Ministry of Justice (MoJ).^3,4^ Discharge can also be granted by the Mental Health Tribunal.^5^ When restricted patients no longer meet the criteria for detention in hospital under the MHA, they are discharged either absolutely or conditionally. Conditionally discharged patients remain subject to controls via the MoJ, including the power of the Secretary of State for Justice to recall them to hospital (under s42 of the MHA) if they need to be detained for treatment.

At the end of 2019, there were 4899 patients detained in hospital under s37/41 in England and Wales, with a further 2821 conditionally discharged in the community.^6^ The vast majority of restricted in-patients are treated in secure services specifically designed to provide a rehabilitative environment.^6,7^ In a small minority of cases, patients are recalled to acute, non-secure in-patient settings. Rather than provide longer-term rehabilitation, acute wards aim to achieve clinical stability and discharge to community services more rapidly. Having restricted patients on acute wards poses particular challenges to services as well as to the patients themselves, primarily due to the additional role of the MHCS.

## Managing Restricted Patients in Non-secure Services

For all restricted patients, whether on secure or acute psychiatric units, the responsible clinician must seek consent via the MoJ to allow a patient to be granted any s17 leave, transfer or discharge. A written application is made to the MHCS, and the decision is made by experienced caseworkers who carry out a risk assessment which considers the clinical opinion of the treating team as well as the patient's offending history and public protection.^8^ In 2019, the MoJ released target times to respond to requests for hospital transfer from the date of application: 28 days for escorted leave, 35 days for unescorted leave and overnight leave, and 28 days for conditional discharge.^9^ These targets are aimed at secure services which manage patients who require rehabilitation. However, in non-forensic services, the time lag between the initial request by the responsible clinician and the authorisation by the Secretary of State for Justice can significantly lengthen the duration of stay for restricted patients when compared with their non-restricted peers on acute wards. This, in turn, can cause frustration for the service and the patient, particularly as patients on acute wards have limited access to occupational and psychological therapy. These frustrations must be balanced with the risk of serious harm that these patients pose to the public.

The aim of this study was to assess the effects of MoJ decision-making on the length of in-patient admissions for s37/41 patients recalled to acute, non-secure services, and their clinical, ethical and resource implications.

## Method

Data were collected from a general adult, non-secure psychiatric hospital in South London and Maudsley NHS Foundation Trust (SLaM). The unit consists of eight wards:

- one 10-bed male psychiatric intensive care unit;
- two 18-bed male acute wards;
- two 16-bed female acute wards;
- one 18-bed female acute ward;
- one 18-bed mixed-sex older-adults ward;
- one 24-bed mixed rehabilitation ward.

The study used data collected as part of a local audit which assessed in-patient admissions according to the Inpatient Care Process Model standards.^10^ Patients who were recalled from conditional discharge were identified from a database of all patients admitted to the hospital under the MHA maintained by the unit's MHA office.

An admission was included in the study if it met the following criteria:

- it was an admission of a recalled patient under s42 of the MHA;
- the patient was admitted after 1 January 2012;
- the patient was discharged before 31 December 2019;
- the admission lasted at least 1 month;
- there was documentation of at least one request and response from the MoJ.

If a patient had two or more admissions during the study period, each admission was included separately. Patients who were initially admitted voluntarily but later subject to recall under s42 were also included.

Demographic and clinical information were extracted from electronic health records by a psychiatrist (G.S.). Extracted data included age, gender, psychiatric diagnosis (assigned by SLaM staff according to ICD-10 criteria), reason for recall and date of admission. The number of days between the request for and granting of escorted leave, unescorted leave, overnight leave, transfer to another ward and discharge to community care were calculated. Instances where requests for leave were rejected by the MoJ and the reasons for such decisions were recorded, as was the method of discharge, i.e. by the Mental Health Tribunal or the MoJ. Data were still extracted if a patient was transferred to another ward at a different hospital within SLaM. If the MoJ rescinded a patient's leave, data on subsequent requests for the same type of leave were also included. Assuming that each day waiting for a response from the MoJ led to an equivalent extension in admission length, we calculated the total additional cost per admission based on a 2019 estimate of the cost of an acute psychiatric bed (£432/day).^11^

### Ethical approval

The data were obtained from an audit completed at SLaM and approved according to local procedures and by the Trust's Caldicott Guardian.

## Results

We identified 14 admissions for patients recalled under s42 of the MHA from conditional discharge in the community to the unit during the study period; two were excluded owing to the admission being shorter than 1 month, and one lacked data on leave requests and responses from the MoJ. No patients were admitted to the ward for older adults. The demographic and clinical characteristics of the study population are described in Table 1. A more detailed description of each admission is given in Table 2. Owing to the small number of subjects, the results are restricted to ensure anonymity.

**Table 1:** Demographic and clinical characteristics of the study population

	*N* (%)
Age, years	
<35	1 (9%)
35–44	3 (27%)
45–65	7 (64%)
Gender	
Male	9 (82%)
Female	2 (18%)
Diagnosis	
Schizophrenia	8 (73%)
Schizoaffective disorder	3 (27%)
Admitted to a SLaM rehabilitation ward during this admission	
Yes	2 (18%)
No	9 (82%)
Admitted to a SLaM forensic ward during this admission	
Yes	1 (9%)
No	10 (91%)
Admitted to SLaM psychiatric intensive care unit ward during this admission	
Yes	8 (73%)
No	3 (27%)
Method of discharge	
MoJ granted	2 (18%)
MHA tribunal	6 (55%)
Transfer to forensic in-patient services	3 (27%)

**Table 2:** Admission pathway and waits

Admission	Admitted from	Escorted leave wait, days	Unescorted leave wait, days	Overnight leave wait, days	Internal transfer wait, days	Discharge wait, days	Total wait, days	Mode of discharge	Discharged destination
1	Rehabilitation unit	NR	30	NR	3	NR	33	Transfer	Medium secure
2	Community	8	59	23	NR	NR	90	Tribunal	Community
3	Community	NR	23	22 + 56	NR	NR	101	Tribunal	Community
4	Community	21	21 + 14	34	14 + 55	NR	159	Tribunal	Community
5	General hospital	63	NR	NR	NR	NR	63	Tribunal	Community
6	Community	ND	28	28	22 + 42	55	175	MoJ approved	Community
7	Medium secure unit	NR	NR	37 + 70	NR	NR	107	Tribunal	Community
8	Community	14	36	NR	NR	NR	50	Transfer	Low secure
9	Community	35	26	26	NR	NR	87	Tribunal	Community
10	Community	35	NR	NR	35	NR	70	Transfer	Low secure
11	Community	8	20	20 + 11	26	22	107	MoJ approved	Community

ND, not documented; NR, not requested.

A total of 26 requests for s17 leave were made to the MoJ, of which 23 were granted, two were rejected and one was not responded to. The mean time to response from the MoJ was 30 days (s.d. 17). Escorted leave was requested seven times (for seven out of 11 admissions), with a mean response time of 26 days (s.d. 20). No requests were rejected by the MoJ. Unescorted leave was requested nine times (eight out of 11 admissions), with a mean response time of 29 days (s.d. 13). Two requests were rejected by the MoJ, one because the MoJ advised that relapse and prevention work was needed (this patient was subsequently transferred to a forensic unit to receive this treatment). The other had no reason documented. There were ten requests for overnight leave for seven admissions. The mean length of time between request and response was 33 days (s.d. 18). No requests were rejected, but one request was not responded to by the MoJ.

Five patients were transferred to another ward during their stay, arising from seven requests to the MoJ for transfers. All transfers were approved. The mean time to response from the MoJ for approval of the transfers was 28 days (s.d. 17).

Six out of eight admissions with a documented discharge pathway were discharged via tribunal, while two were approved for discharge by the MoJ. The mean time to response from the MoJ for approval of discharge was 38 days (s.d. 23). No requests for discharge were declined by the MoJ. Two admissions were transferred out of SLaM, and one admission had no clearly documented discharge pathway.

The mean total wait for response from the MoJ for requests for s17 leave, transfers and discharges overall was 95 days per admission (range 33–175; s.d. 43). At a cost of £432/bed day, the estimated mean cost of wait per admission was £40 922.

## Discussion

This study found that patients recalled to hospital from conditional discharge in the community experience considerable periods of waiting for the MoJ to grant leave, transfer and discharge during admissions to acute, non-secure in-patient wards owing to their status as restricted patients. To our knowledge, this is the first study to examine patients in these unique circumstances. Although the size of our sample may have been small, our results highlight an important clinical issue with ethical, clinical and resource implications.

Secure services aim to provide long-term management for patients with complex needs who present a significant level of risk.^8^ They provide long-term rehabilitation through intensive multidisciplinary treatment from a wide range of professionals and greater recreational support than that provided in acute, non-secure services.^8^ Acute, non-forensic services, on the other hand, provide support for patients during an acute illness, with a median length of stay less than 1 month.^12^ They often lack psychological or occupational therapy support and are built for crisis management rather than rehabilitation. This raises the question as to whether it is appropriate for conditionally discharged patients to be recalled to acute units.

Patients recalled from a conditional discharge in the community to acute services wait for weeks at a time for a response from the MoJ on wards that do not provide these specialist services. On the other hand, admitting a patient into secure services merely because of their restricted status, where the risk to others may not warrant such a high level of input, may be too restrictive. This current practice, illustrated by the data in this study, is at odds with the principles of the MHA, which emphasise the importance of least restrictive practices while maximising the independence of the patient, as well as offering care and treatment that is appropriate to the patient's needs and can facilitate timely and safe discharge.^13^ It is of further note that in our study, the MoJ did not deem specialist services to be essential for the patient's treatment pathway for ten out of 11 admissions. Only in one case did the MoJ reject a request for leave; this was because the patient had not completed relapse prevention work, something that is not normally offered within acute services, and resulted in the patient being transferred to a forensic unit. This then raises the question of whether recall is an appropriate option when admitting such patients to acute services in the first place, compared with an informal admission or detention under Part 2 of the MHA, which do not require MoJ input for leave and discharge decisions.

Clinicians therefore need to carefully consider all options and liaise with the MoJ accordingly before considering recall of such patients. If a patient is admitted to an acute bed rather than a secure one, it suggests that the patient's mental state and risks do not require the level of security and complex management provided by secure services, even if they are on a s41. Use of a civil section would avoid the non-clinical waits of response from the MoJ, as the responsible clinician would be able to facilitate s17 leave and discharge without MoJ input. The MoJ provides guidance on when such patients can be admitted to hospital either informally or under a civil section. The guidance states that admissions under civil sections of the MHA or informally should be considered where the admission is likely to be no longer than several weeks and where the main concern is risk to self with no evidence of risk to others.^14^ Put simply, if restricted patients can be managed in acute, non-secure services then perhaps they should be treated as such.

The complicating factor is the issue of risk. Patients placed on s41 of the MHA are by definition deemed to be at risk of serious harm to the public.^15^ This raises the question of whether the use of civil sections or informal admissions are ever suitable for such high-risk patients. When assessing a patient's leave or discharge, the MoJ looks specifically at the level of risk to the public and measures that are in place to alleviate this risk,^16^ aims that may overlap with the responsible clinician's judgement but are ultimately more focused on public protection than on the care and treatment of the patient.^17^ This balance between public protection and treatment of the individual patient can be difficult to get right. An argument could be made that a range of professionals can offer a variety of perspectives on the issues of risk, e.g. a clinical perspective of risk can come from the psychiatrist while a more criminological perspective of risk is provided by the MoJ.^16^ General adult psychiatrists may have less experience in dealing with such patients and may prefer MoJ input as it provides a more systematic overview of risk management.^18^ Some have welcomed input from the MoJ, referring to it as an external audit, while others have commented on the collective experience of the MoJ in dealing with restricted patients, which can be helpful to the lone psychiatrist.^15^ On the other hand, as our data show, recalled restricted patients on acute wards experience prolonged stays and potential delays in being granted leave. What is not clear is whether this is because they present a significant risk to the public at that point in time, or whether it is purely an artefact of leave needing to be approved by the MoJ.

The 2018 Independent Review of the MHA commented on the significant waits experienced by restricted patients being transferred, noting that the majority of requests were granted by the MoJ anyway, thereby raising the question of why the clinical team must go through this long process in the first place.^19^ It is of note that the Review's recommendation to allow the responsible clinician to make the decision for a restricted patient to be transferred if the decision were to carry little risk^19^ was rejected in the UK government's White Paper on the basis that such waits were being managed by the MHCS via their targets set in 2019.^20^ The question is whether these targets are suitable for such patients within acute in-patient settings, where expectations of patient management are very different.

### A human rights perspective on the restriction order

Supporting human rights is an essential part of mental healthcare. Deprivation of liberty is a significant breach of an individual's autonomy and can only be conducted in clearly defined and exceptional circumstances.^21^ The main piece of international legislation that covers the remits of deprivation of liberty in the UK is the European Convention of Human Rights (ECHR). Article 5(1) of the ECHR states that individuals can only be deprived of their liberty under a procedure prescribed by law,^22^ while Article 5(1)(e) allows the detention of individuals who are of ‘unsound mind’.^23^ This was clarified in the Winterwerp case,^24^ which outlined three criteria that must be met before an individual of unsound mind can be lawfully deprived of their liberty: (a) Objective medical expertise to confirm the presence of unsound mind; (b) mental disorder of a kind or degree requiring confinement; and (c) persistence of confinement only based on the ongoing presence of the mental disorder. Yet the results of our study suggest that patients under s37/41 of the MHA continue to be deprived of their liberty even after the responsible clinician has stated they are ready for leave or discharge. Thus, the waits experienced by patients in this study resulted in deprivation of liberty which did not meet the Winterwerp criteria, i.e. there was no medical expertise supporting the prolonged confinement of these patients within psychiatric hospitals. Furthermore, these waits were not a result of medical decision-making but of delays in process and communications by non-medical professionals. One could argue that such waits could breach Article 5(1) of the ECHR and are a breach of the individual's right to liberty. It is to be noted that these waits are not insignificant when one considers the typically short length of stay in acute services; there is a question as to whether it is reasonable for the MoJ to take several weeks on average to respond to requests for leave and discharge which have already been deemed appropriate by a medical professional. Despite the government's response in the White Paper that such timeframes have decreased following the introduction of targets in 2019,^20^ the question remains as to whether the targets of response in 28 days for escorted leave and 35 days for unescorted leave^9^ are acceptable in a setting that is not built for long-stay and complex patients. Although we acknowledge the potential risks that these patients may present to the public and thus the desire for detailed risk assessments, the limited predictive value of such assessments must also be considered.^25^

These prolonged waits for responses from the MoJ can have psychological consequences as well as legal ones. Studies of restricted patients in secure services have explored the sense of loss of control and inferiority resulting from waits for response from the MoJ, as well as concerns around institutionalisation.^26^ One study found that the majority of participants voiced frustrations about waiting for the MoJ to respond to requests for leave, feeling that they were a case rather than a patient.^26^ In another qualitative study involving interviews with professionals working with restricted patients in a private hospital, concerns were raised by staff about the MoJ placing too much emphasis on the wording of the MHA and not enough on the individual patient's circumstances.^16^ This perception of cautiousness and overemphasis on risk by the MoJ has been noted in other studies.^16,17,26^ These issues are likely to be aggravated on acute, non-secure wards, as patients spend months confined in an environment which is not designed to support long-term admissions.

### Resource implications

A final consideration is the resource implications for acute mental health trusts when patients are awaiting responses from the MoJ. The mean total wait per admission was 95 days, which was estimated to cost the hospital trust £40 922 per admission based on estimated costs from 2019. We assumed that each day waiting for a response from the MoJ led to an equivalent extension of admission length. This may not be the case, as some clinicians may have pre-emptively requested leave or discharge in anticipation of long wait times. In addition, there may have been a knock-on effect where a long wait for granting of leave may have brought forward, at least relatively, a patient's readiness for discharge. It is important to note that this cost only applies to acute services, as the cost of secure beds is significantly higher.^11^ The budget for UK mental health services has been under considerable strain over recent years,^27,28^ and the number of psychiatric beds available has been steadily falling.^29^ With rising pressures on psychiatric beds in the country, it is unclear why budgets assigned for the clinical management of acute psychiatric illness should be used to contain patients with more complex needs who require specialist management.

### Strengths and limitations

The strengths of this study include the use of a systematically recorded database maintained by the local MHA office to identify all patients who were detained under s37/41 after being recalled from conditional discharge. The study was limited by the use of clinical notes, which are prone to entry errors by clinicians. In particular, there may have been instances where waits for MoJ responses to requests for leave, transfer and discharge were incurred but not recorded, leading to underestimation of waits and costs.

The population included in this study was demographically representative of the overall restricted patient population, which is noted to be mostly male, aged 35–55 years, and discharged by a MHA tribunal rather than the MoJ.^5^ However, our sample was a unique population – restricted patients treated in acute, non-secure services – and was not representative of the wider restricted patient population, the majority of whom are treated in secure services.^7^ The reliance on a sample from a single hospital may also limit generalisation of our findings.

### Future perspectives

Patients recalled from the community to general adult, non-secure wards under s42 of the MHA experience considerable waits for response from the MoJ. The clinical, ethical and resource implications of these waits deserve further consideration. There are at least three possible solutions to this issue. The first is that, as suggested by the 2018 Independent Review of the MHA, responsible clinicians could be allowed to make low-risk decisions for restricted patients, with the MoJ having the ability to object. Although we acknowledge that the UK government has rejected this recommendation on the basis that MoJ responses to requests have improved following the initiation of targets in 2019, the question still remains as to whether such targets are appropriate for restricted patients within acute settings. The second is that, for patients managed in non-secure services, the MoJ must approve or reject requests for leave, transfer and discharge within a shorter timeframe. The third is that practice and guidance on the use of voluntary admissions and civil sections should be amended such that recall is only used as a last resort. When conditionally discharged patients are deemed suitable for admission to acute, non-secure wards, the responsible clinician and the MoJ can continue to liaise about the patient's risks without the need for recall unless it is deemed absolutely necessary. This would arguably better support patient rights and uphold the guiding principles of the MHA, namely using the least restrictive option and maximising independence; purpose and effectiveness; respect and dignity; empowerment and involvement; and, of particular significance here, efficiency and equity.

Each of our suggested options could significantly shorten restricted patients’ length of stay and the subsequent effects on their mental health and rights while maintaining MoJ oversight of the risks these patients present to the public.

## Data Availability

The full data from this audit are not publicly available as they may identify the participants given the small sample size.

## About the authors

**Gunjan Sharma** is a Core Psychiatry Trainee at the South London and Maudsley NHS Foundation Trust, London, UK; **Penelope Brown** is a Consultant Forensic Psychiatrist at the South London and Maudsley NHS Foundation Trust, London, UK, and Department of Psychological Medicine, Institute of Psychiatry, Psychology & Neuroscience, King's College London, London, UK; **Ijaz Ur Rehman** is a Consultant Psychiatrist at the South London and Maudsley NHS Foundation Trust, London, UK; and **Edward Chesney** is a Specialty Registrar at the South London and Maudsley NHS Foundation Trust, London, UK, and Department of Psychosis Studies, Institute of Psychiatry, Psychology & Neuroscience, King's College London, London, UK.
